# Patterns of spatial genetic structures in *Aedes albopictus* (Diptera: Culicidae) populations in China

**DOI:** 10.1186/s13071-019-3801-4

**Published:** 2019-11-21

**Authors:** Yong Wei, Jiatian Wang, Zhangyao Song, Yulan He, Zihao Zheng, Peiyang Fan, Dizi Yang, Guofa Zhou, Daibin Zhong, Xueli Zheng

**Affiliations:** 1 Department of Pathogen Biology, School of Public Health, Southern Medical University, Guangzhou, China; 2 Program in Public Health, College of Health Sciences, University of California, Irvine, USA

**Keywords:** *Aedes albopictus*, Microsatellite, Genetic diversity, Population structure, Gene flow, Dengue

## Abstract

**Background:**

The Asian tiger mosquito, *Aedes albopictus*, is one of the 100 worst invasive species in the world and the vector for several arboviruses including dengue, Zika and chikungunya viruses. Understanding the population spatial genetic structure, migration, and gene flow of vector species is critical to effectively preventing and controlling vector-borne diseases. Little is known about the population structure and genetic differentiation of native *Ae. albopictus* in China. The aim of this study was to examine the patterns of the spatial genetic structures of native *Ae. albopictus* populations, and their relationship to dengue incidence, on a large geographical scale.

**Methods:**

During 2016–2018, adult female *Ae. albopictus* mosquitoes were collected by human landing catch (HLC) or human-bait sweep-net collections in 34 localities across China. Thirteen microsatellite markers were used to examine the patterns of genetic diversity, population structure, and gene flow among native *Ae. albopictus* populations. The correlation between population genetic indices and dengue incidence was also examined.

**Results:**

A total of 153 distinct alleles were identified at the 13 microsatellite loci in the tested populations. All loci were polymorphic, with the number of distinct alleles ranging from eight to sixteen. Genetic parameters such as PIC, heterozygosity, allelic richness and fixation index (*F*_ST_) revealed highly polymorphic markers, high genetic diversity, and low population genetic differentiation. In addition, Bayesian analysis of population structure showed two distinct genetic groups in southern-western and eastern-central-northern China. The Mantel test indicated a positive correlation between genetic distance and geographical distance (*R*^2^ = 0.245, *P* = 0.01). STRUCTURE analysis, PCoA and GLS interpolation analysis indicated that *Ae. albopictus* populations in China were regionally clustered. Gene flow and relatedness estimates were generally high between populations. We observed no correlation between population genetic indices of microsatellite loci in *Ae. albopictus* populations and dengue incidence.

**Conclusion:**

Strong gene flow probably assisted by human activities inhibited population differentiation and promoted genetic diversity among populations of *Ae. albopictus*. This may represent a potential risk of rapid spread of mosquito-borne diseases. The spatial genetic structure, coupled with the association between genetic indices and dengue incidence, may have important implications for understanding the epidemiology, prevention, and control of vector-borne diseases.

## Background

*Aedes* (*Stegomyia*) *albopictus* (Skuse, 1894) originated in Southeast Asia and spread to several islands in the Pacific and the Indian Oceans during the 17th and 18th centuries [1]. It was discovered in Albania in Europe in 1979 [2], the USA and Brazil in North and South America in 1986 [3, 4], Fiji in Oceania in 1990 [5] and Nigeria in Africa in 1992 [6]. Global warming, winter diapause, and human-aided transportation have contributed to the global invasion of *Ae. albopictus* [7–9].

As a vector of over 20 arboviruses [10] and one of the 100 worst invasive species in the world [11], *Ae. albopictus* poses serious public health concerns for arbovirus-related disease outbreaks. In China, dengue has been a threat for 40 years, since the first reported outbreak in 1978 [12]. Local dengue transmission has been identified in Guangdong, Guangxi, Hainan, Yunnan, Fujian, Zhejiang and Henan provinces [13]. *Aedes albopictus* and *Ae. aegypti* are two important vector species responsible for dengue transmission in China. *Aedes albopictus* is the most predominant species and has been found in nearly one third of China [14]. This species has a wide range of distribution from north of 41°N latitude to the southern reaches of the country, while the distribution of *Ae. aegypti* is limited to small areas of southern China, including Hainan, Guangdong, Guangxi and Yunnan provinces [15, 16]. Li et al. [17] reported that *Ae. aegypti* was found only in two sites (Maoming, Guangdong and Sanya, Hainan) of the 26 mosquito surveillance sites in China. The relative proportion of *Ae. albopictus* to *Ae. aegypti* was > 20:1 in the two sites during 2005–2015, while the proportion of *Ae. albopictus* to *Ae. aegypti* was approximately 1:2 in 2014 in Jinghong city, Yunnan Province [18]. *Aedes albopictus* has been reported to be the sole mosquito vector for dengue transmission in Guangzhou, with no presence of *Ae. aegypti* identified over the past three decades [19]. The strong adaptability of *Ae. albopictus* has contributed to the reemergence and wide spread of dengue [20] and the outbreaks of mosquito-borne diseases have spread with the expansion of mosquito habitats [21]. Vector surveillance, dispersal monitoring, and control play an important role in controlling outbreaks [22].

Molecular markers can provide abundant information that is critical to studying the sources and routes of vector invasions [11, 23]. As the second generation of DNA markers, microsatellites or simple sequence repeats (SSRs), consisting of short tandem repeats of 1–6 nucleotides, are widely distributed in the genomes of animals and plants [24]. Due to the advantages of simple operation, easy detection, good reproducibility, high polymorphism, and the inheritance of Mendelian codominance [25], microsatellite markers have been utilized to evaluate genetic diversity and population structure, and identify invasions of *Ae. albopictus* [22, 26, 27]. Multiple microsatellite markers for *Ae. albopictus* have been isolated previously, and there are more than 50 microsatellites applied in population genetic studies of *Ae. albopictus* [11]. Although mitochondrial DNA markers are rapidly evolving, non-recombining, maternally inherited, and exhibit high rates of mutation, mtDNA is not a strictly neutral marker; strong directional selection on mtDNA sequence has been reported in several insect species [28–30]. In order to obtain more accurate and detailed information on genetic structure, large-scale multi-site samplings should be conducted, and neutral microsatellite markers should be used to investigate *Ae. albopictus* population genetics.

The present study was designed to address the following questions: (i) What is the level of genetic diversity and population differentiation in and between *Ae. albopictus* populations in China?; (ii) What are the patterns of colonization and dispersal (or gene flow) of *Ae. albopictus* in China?; (iii) Is there any association between genetic diversity indices and local dengue incidence? To address these questions, extensive mosquito samplings and a set of thirteen microsatellite loci were used in the study. The data gained from this study could provide useful information for understanding the epidemiology, prevention, and control of vector-borne diseases.

## Methods

### Mosquito sampling and DNA extraction

**Fig. 1:**
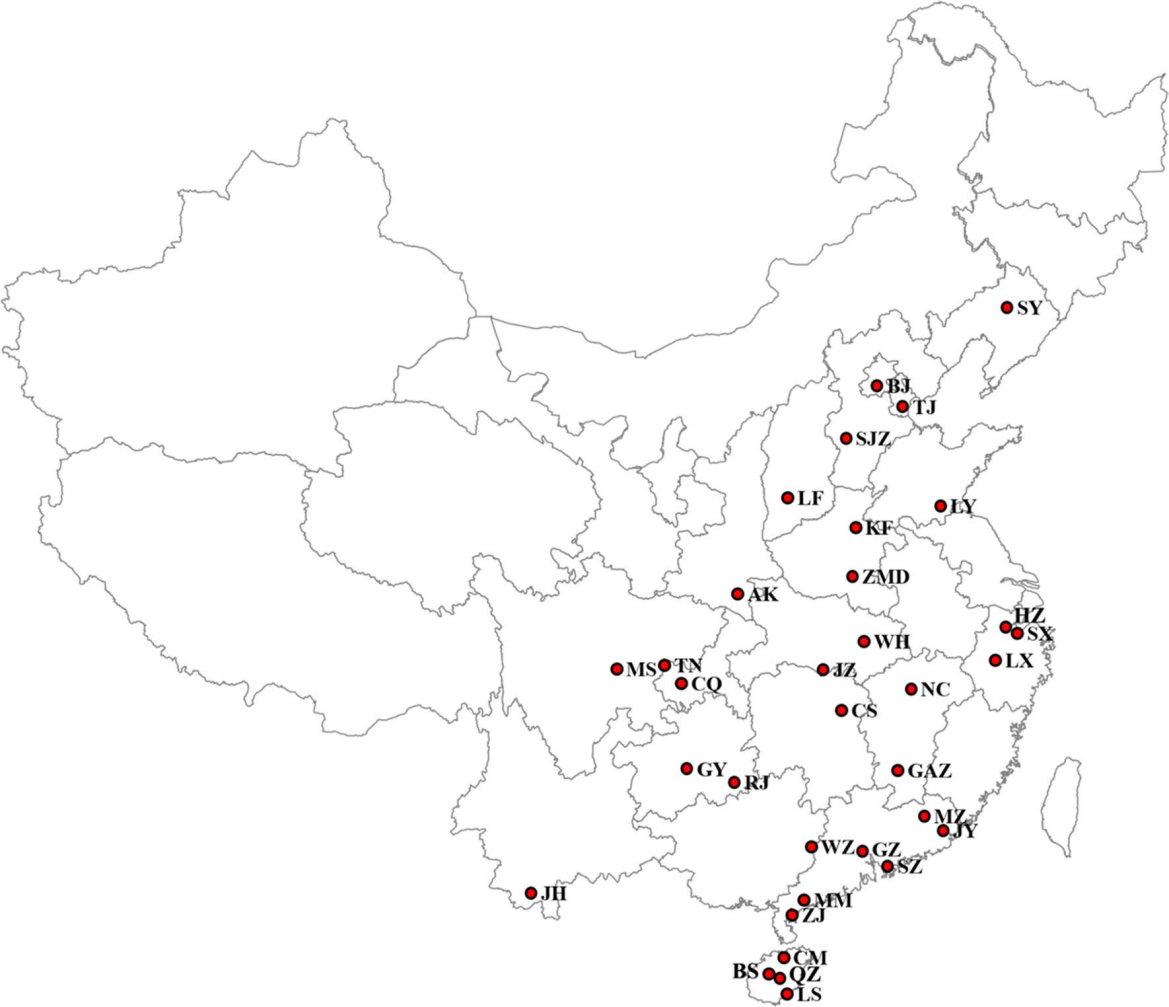
Geographical locations of *Ae. albopictus* sampling sites in China. *Abbreviations*: LS, Lingshui; QZ, Qiongzhong; BS, Baisha; CM, Chengmai; ZJ, Zhanjiang; MM, Maoming; SZ, Shenzhen; GZ, Guangzhou; JY, Jieyang; MZ, Meizhou; JH, Jinghong; WZ, Wuzhou; RJ, Rongjiang; GY, Guiyang; CQ, Chongqing; TN, Tongnan; MS, Meishan; CS, Changsha; GAZ, Ganzhou; NC, Nanchang; WH, Wuhan; JZ, Jingzhou; AK, Ankang; LX, Lanxi; SX, Shaoxing; HZ, Hangzhou; ZMD, Zhumadian; KF, Kaifeng; LY, Linyi; LF, Linfen; SJZ, Shijiazhuang; TJ, Tianjin; BJ, Beijing; SY, Shenyang

From 2016 to 2018, adult female *Ae. albopictus* mosquitoes were collected by human landing catch (HLC) or human-bait sweep-net collections in 34 locations in 19 provinces across northern and southern China, including Hainan Island (Fig. 1, Table 1). The abbreviations of the 34 locations are as follows: LS, Lingshui; QZ, Qiongzhong; BS, Baisha; CM, Chengmai; ZJ, Zhanjiang; MM, Maoming; SZ, Shenzhen; GZ, Guangzhou; JY, Jieyang; MZ, Meizhou; JH, Jinghong; WZ, Wuzhou; RJ, Rongjiang; GY, Guiyang; CQ, Chongqing; TN, Tongnan; MS, Meishan; CS, Changsha; GAZ, Ganzhou; NC, Nanchang; WH, Wuhan; JZ, Jingzhou; AK, Ankang; LX, Lanxi; SX, Shaoxing; HZ, Hangzhou; ZMD, Zhumadian; KF, Kaifeng; LY, Linyi; LF, Linfen; SJZ, Shijiazhuang; TJ, Tianjin; BJ, Beijing; and SY, Shenyang.

**Table 1:** Sampling information of *Ae. albopictus* collection in China

Province	Sample sites	Abbreviation	Sample size	Latitude	Longitude	Collection date
Hainan	Lingshui	LS	32	18°30ʹ27″N	110°01ʹ59″E	August 2016
	Qiongzhong	QZ	32	19°02ʹ06″N	109°50ʹ03″E	August 2016
	Baisha	BS	22	19°13ʹ37″N	109°26ʹ51″E	August 2016
	Chengmai	CM	32	19°44ʹ25″N	110°00ʹ02″E	September 2016
Guangdong	Zhanjiang	ZJ	32	21°05ʹ37″N	109°42ʹ60″E	September 2018
	Maoming	MM	28	21°31ʹ55″N	111°0ʹ31″E	August 2018
	Shenzhen	SZ	32	22°32ʹ26″N	113°59ʹ56″E	September 2018
	Guangzhou	GZ	32	23°11ʹ15″N	113°19ʹ42″E	June 2018
	Jieyang	JY	32	23°37ʹ43″N	116°16ʹ43″E	August 2018
	Meizhou	MZ	32	24°08ʹ11″N	115°40ʹ26″E	August 2018
Yunnan	Jinghong	JH	25	22°0ʹ32″N	100°48ʹ0″E	April 2018
Guangxi	Wuzhou	WZ	33	23°22ʹ51″N	110°54ʹ58″E	October 2018
Guizhou	Rongjiang	RJ	32	25°56ʹ31″N	108°31ʹ40″E	August 2018
	Guiyang	GY	31	26°33ʹ24″N	106°45ʹ36″E	August 2018
Chongqing	Chongqing	CQ	32	29°30ʹ32″N	106°28ʹ36″E	June 2018
	Tongnan	TN	28	30°09ʹ59″N	105°49ʹ54″E	June 2018
Sichuan	Meishan	MS	32	30°11ʹ55″N	103°52ʹ01″E	September 2018
Hunan	Changsha	CS	32	28°14ʹ25″N	113°04ʹ15″E	July 2018
Jiangxi	Ganzhou	GAZ	32	25°52ʹ18″N	115°01ʹ29″E	September 2018
	Nanchang	NC	30	28°40ʹ54″N	115°54ʹ27″E	July 2018
Hubei	Wuhan	WH	31	30°30ʹ30″N	114°22ʹ39″E	July 2018
	Jingzhou	JZ	28	29°50ʹ11″N	112°28ʹ15″E	July 2018
Shanxi (west)	Ankang	AK	18	32°36ʹ21″N	108°25ʹ57″E	September 2018
Zhejiang	Lanxi	LX	32	29°13ʹ16″N	119°28ʹ35″E	October 2018
	Shaoxing	SX	27	29°50ʹ51″N	120°30ʹ04″E	August 2018
	Hangzhou	HZ	25	30°18ʹ42″N	120°07ʹ09″E	August 2018
Henan	Zhumadian	ZMD	32	32°58ʹ34″N	114°0ʹ27″E	August 2018
	Kaifeng	KF	32	34°47ʹ53″N	114°18ʹ05″E	August 2018
Shandong	Linyi	LY	31	35°20ʹ18″N	118°09ʹ06″E	August 2018
Shanxi (east)	Linfen	LF	32	36°10ʹ34″N	111°36ʹ01″E	September 2018
Hebei	Shijiazhuang	SJZ	32	37°54ʹ55″N	114°27ʹ49″E	August 2018
Tianjin	Tianjin	TJ	32	39°06ʹ19″N	117°10ʹ41″E	August 2018
Beijing	Beijing	BJ	30	39°51ʹ36″N	116°11ʹ45″E	August 2018
Liaoning	Shenyang	SY	28	41°52ʹ28″N	123°33ʹ36″E	August 2018

In each location, 8–12 households or collection points at 400–3000 m apart were selected randomly for adult mosquito collections to minimize sibling bias. Approximately 30 *Ae. albopictus* female mosquitoes from each location with 2–3 individuals per collection point were used for DNA extraction and genetic analysis. Local dengue surveillance data from the 19 provinces during 2011–2015 were obtained from the website (http://www.phsciencedata.cn/Share/en/index.jsp) of the Public Health Science Data Center managed by the Chinese Center for Disease Control and Prevention. The 19 provinces include those with frequent dengue fever outbreaks in the past 30 years, including Guangdong, Guangxi, Yunnan, Zhejiang and Hainan, as well as provinces with dengue outbreaks in recent years, including Hubei, Hunan, Guizhou and Jiangxi. Other provinces have imported cases, including Shanxi, Shandong and Liaoning. The collected specimens were identified morphologically to *Ae. albopictus*, and samples were stored in 70% ethanol. Total genomic DNA was individually extracted using the Insect DNA Kit (Omega Bio-tek, GA, USA), following the manufacturer’s protocol.

### Cryptic species identification and *Wolbachia* infection

*Aedes albopictus* cryptic species identification was performed by DNA sequencing of the mitochondrial gene cytochrome *c* oxidase subunit 1 (*cox*1) for the samples collected in Guangxi Province, where the cryptic species have been detected previously [31]. The *Wolbachia* infection status of individual mosquitoes was examined by PCR amplification of *Wolbachia* ribosomal DNA using a previously published method [31].

### Microsatellite genotyping

A set of thirteen microsatellite markers was used to examine the patterns of genetic diversity, population structure, and gene flow among the native mosquito populations. These markers, which were developed in previous studies, included 3 dinucleotide and 10 trinucleotide loci [27, 32]. The PCR reaction mixture consisted of 40 ng genomic DNA, 7.5 μl 2× PCR Master Mix (Promega, Madison, WI, USA), 0.05 μl (10 μM) M13 tagged forward primer, 0.2 μl (10 μM) reverse primer, and 0.2 μl M13 tagged fluorescent dye (FAM, HEX or TAMRA), in a final volume of 15 μl. The PCR cycling conditions were as follows: 95 °C for 5 min; 30 cycles of 95 °C for 30 s, 58 °C for 40 s, and 72 °C for 45 s; 8 cycles of 95 °C for 30 s, 53 °C for 40 s, and 72 °C for 45 s; and a final extension at 72 °C for 10 min. PCR products were sent to the Beijing Genomics Institute (BGI, Shenzhen, China) and processed in an automatic sequencer ABI 3730 (Applied Biosystems, Foster City, CA, USA). Allele sizes for each locus were read with GeneMarker software (version 2.6.3) [33].

### Data analysis

#### Analysis of pairwise relatedness

Pairwise genetic relatedness between individuals was examined by LRM estimator [34] and QGM estimator [35] using GenAlex v.6.5 [36] to detect kinship within and between *Ae. albopictus* populations. The coefficient of relationship, *r* ≥ 0.25 for LRM and *r* ≥ 0.5 for QGM was used to define a full sibling relationship (parents and offspring, or siblings that share the same parents), and a value of 0.125 < *r* < 0.25 for LRM and 0.25 < *r* < 0.5 for QGM indicates a half sibling (one shared parent). In order to reduce the siblings bias within population for analysis of genetic structure, we selected only one individual from each putative full-sibling group within any population.

#### Genetic diversity and differentiation

Genetic variation within each locality was estimated in terms of the average number of alleles (*Na*), effective number of alleles (*Ne*), inbreeding coefficient (*F*_IS_), fixation index (*F*_ST_), observed heterozygosity (*Ho*) and expected heterozygosity (*He*) using GenAlEx v.6.5 [36]. The number of migrants per generation, or gene flow, was calculated using the following formula: *Nm* = (1/*F*_ST_ − 1)/4 [37]. Polymorphic information content (PIC) across all sixteen loci was assessed using Microsatellite Toolkit [38]. The possible presence of null alleles was checked at a population level for each marker using Micro-Checker (version 2.2.3) [39]. To determine whether the individuals in each study location were sufficient for the research, the mean number of alleles per locus (*Na*) for each population was calculated with a rarefaction method using Allelic Diversity Analyzer (version 1.0) [40]. Deviation from Hardy–Weinberg equilibrium (HWE) and linkage disequilibrium (LD) were computed using GENEPOP version 4.7 [41, 42]. Analysis of molecular variance (AMOVA) was performed using Arlequin version 3.5.2.2 [43]. To detect bottlenecks, the Wilcoxon test for heterozygosity excess was conducted with a two-phase mutation (TPM, 70% proportion of stepwise-mutation model in TPM, 30% variance for TPM, 1000 iterations) in BOTTLENECK v.1.2.02 [44, 45]. Genetic landscape shape (GLS) interpolation analysis was conducted using Alleles In Space (AIS) software based on the pairwise genetic and geographical distance matrices [46]. Results of GLS interpolation analysis were imported into Surfer software to produce the contour map [47]. A Mantel test on geographical and genetic distance (pairwise phiPT) was performed in GenAlEx v.6.5 using 9999 permutations. The correlation between geographical and genetic distance was plotted and the correlation coefficient (*r*) as well as *R*-squared were estimated using GenAlEx v.6.5.

### Population structure

Geographical population structure was evaluated using the Bayesian clustering method in STRUCTURE v.2.3 [48], which identifies the most probable number of genetic clusters (K) and assigns individuals to these clusters. We conducted different runs using different datasets for further clustering. For each dataset, the most likely number of clusters (K) was determined by conducting 5 independent runs for each K, from K = 1 to the maximum number of populations included in the analysis. Each run assumed an admixture model and independent allele frequencies using a ‘burn-in’ value of 50,000 iterations followed by 200,000 repetitions. The optimal number of clusters (K) was determined using the Delta K method of Evanno et al. [49] in the online version of Structure Harvester v.0.6.94 [50]. Then we used a ‘burn-in’ of 100,000 and runtime of 2,000,000 generations per iteration (20 iterations) for the optimal K-values [26]. To compile data from the 20 iterations for the independent values of K, we used the Greedy algorithm in CLUMPP v.1.1.2 with 1000 replicates [51]. The results were plotted in DISTRUCT v.1.1 [52]. Relationships among populations were assessed using principal coordinates analysis (PCoA) in GenAlEx v.6.5.

### Correlation analysis between genetic indices and dengue incidence

Dengue incidence was calculated as the number of cases per 100,000,000 people. Due to the high variation in the number of cases, the data were rescaled with a log-transformation using the equation Ln (µ), where µ is the number of cases per 100,000,000 people. Pearson’s correlation coefficient was used to analyze the correlation between dengue incidence after transformation and genetic indices, including allelic richness, private allelic richness, effective alleles, Shannon’s information index, observed heterozygosity and inbreeding coefficient. The correlation analysis was performed using SPSS version 22. A significance level at *P* < 0.05 was set for all statistical tests, and the sequential Bonferroni correction [53] was used when significant correlations were detected between the paired data.

## Results

### Detection of *Ae. albopictus* cryptic species and *Wolbachia* infection

No *Ae. albopictus* cryptic species was detected in the samples collected from Guangxi Province. The *cox*1 haplotype was identical with the sequences on GenBank (KY765450, KY765452 and KY765455). All the samples showed positive infection of *Wolbachia* with type A and/or type B. The majority of samples (> 93%) were infected with type A and type B together, only a few samples were infected with type A (5.1%) or type B (1.6%) (Additional file 1: Table S1).

### Microsatellite analysis of genetic variability and diversity

Genotypes at 13 microsatellite loci were determined for 1023 *Ae. albopictus* specimens collected in 34 locations (Table 1). We compared the detailed pairwise relatedness of all specimens within and between populations (Additional file 2: Table S2). Genetic relatedness of full sibling among individuals within population were observed in 16 out of the 34 *Ae. albopictus* populations with high proportion of kinship in Wuzhou, Beijing and Shenyang populations. Full sibling relationships were also found among 32 (94%) of the 34 *Ae. albopictus* populations with the highest proportion of kinship between Beijing and Shenyang. There were 559 pairs (0.107%) of half siblings and 68 pairs (0.013%) of full siblings among individual within populations, and 5882 pairs (1.125%) of half siblings and 82 pairs (0.016%) of full siblings among individuals between populations (Table 2). We excluded 46 samples of full siblings in collection sites and used the data from 977 *Ae. albopictus* specimens to conduct further analysis. In total, 153 alleles were obtained. All loci were polymorphic, showing several distinct alleles ranging from eight (Aealbmic9) to sixteen (ALB-TRI-6) with an average of 11.769 per locus (Table 2). Micro-Checker results suggest that locus Aealbmic12 has a high probability of null alleles, as its estimated null allele frequency is 0.317 calculated by Brookfield’s method [54]. There is no evidence of null alleles for loci Aealbmic9, Aealbmic8, Aealbmic6, ALB-DI-6 and ALB-TRI-33 (Table 2). The remaining loci exhibited signs of having null alleles, but their estimated frequency was lower than 0.200 (Table 2). PIC was high, with values ranging from 0.447 (ALB-DI-4) to 0.846 (ALB-TRI-6) (mean value 0.691) (Table 2). Shannon’s information index was consistent with PIC, ranging from 0.880 (ALB-DI-4) to 1.973 (ALB-TRI-6) (mean value of 1.645) (Table 2). Among the 442 pairs tested for Hardy–Weinberg equilibrium (HWE) at each locus per population site after Bonferroni corrections, 158 pairs significantly departed from HWE (*P* < 0.05), where 146 of these significant departures indicated heterozygosity deficits (Additional file 3: Table S3). No locus-by-locus pair showed significance consistently across all locations, though 165 of 2652 (6.22%) pairs tested for linkage disequilibrium (LD) remained significant (Additional file 4: Table S4). Fifty-three (11.99%) pairs tested for HWE lost the significance after Bonferroni corrections, so did the 161 (6.07%) pairs tested for LD.

**Table 2:** Genetic relatedness between samples in *Ae. albopictus* population

Kinship	*n*	Percentage
Within populations		
Half sibling	559	0.107
Full sibling	68	0.013
Total	627	0.120
Between populations		
Half sibling	5882	1.125
Full sibling	82	0.016
Total	5964	1.141
Total pairwise comparisons	522,753	

*Abbreviation*: n, number of comparisons

The genetic diversity and difference analysis of *Ae. albopictus* showed that the mean number of alleles per locus at each location ranged from 5.308 (SY) to 9.000 (LS), with an average of 6.912, and the allelic richness ranged from 4.327 (SY) to 7.561 (LS), with an average of 5.995 (Additional file 5: Table S5). Allelic richness increased as the sample size increased (Additional file 6: Figure S1). The *Ho* values for each locus ranged from 0.394 (ALB-DI-4) to 0.775 (Aealbmic9), and the *He* values for each locus ranged from 0.456 (Aealbmic6) to 0.824 (ALB-TRI-6) (Table 3). The *Ho* values for each location ranged from 0.501 (QZ) to 0.690 (SZ), and the *He* values for each location ranged from 0.619 (SY) to 0.763 (LS) (Additional file 5: Table S5). Except in the location SZ, the *He* values were higher than the *Ho* values in all locations. Using the TPM model, tests for recent population bottlenecks based on gene diversity and allele frequency distribution were not significant, except in the SJZ population (Additional file 5: Table S5).

**Table 3:** Genetic indices for genetic markers of *Ae. albopictus* from China

Locus	No. of alleles	Estimated null allele frequency	PIC	SI	*Ho*	*He*
Aealbmic9	8	–	0.642	1.309	0.775	0.672
Aealbmic10	9	0.137	0.547	1.051	0.573	0.586
Aealbmic8	11	–	0.816	1.785	0.761	0.809
Aealbmic12	11	0.317	0.819	1.850	0.574	0.812
Aealbmic6	10	–	0.453	0.947	0.462	0.456
Aealbmic5	14	0.108	0.661	1.364	0.570	0.667
Aealbmic16	15	0.102	0.716	1.591	0.599	0.708
ALB-TRI-6	16	0.091	0.846	1.973	0.629	0.824
ALB-DI-6	13	–	0.744	1.569	0.485	0.743
ALB-DI-4	9	0.139	0.447	0.880	0.394	0.466
Aealbmic3	14	0.154	0.815	1.846	0.664	0.791
ALB-TRI-33	10	–	0.722	1.451	0.721	0.721
Aealbmic11	13	0.122	0.752	1.560	0.544	0.744
Mean	11.769		0.691	1.475	0.596	0.692

*Abbreviations*: PIC, polymorphic information content; SI, Shannonʼs index; *Ho*, observed heterozygosity; *He*, expected heterozygosity

### Genetic structure and differentiation

**Fig. 2:**
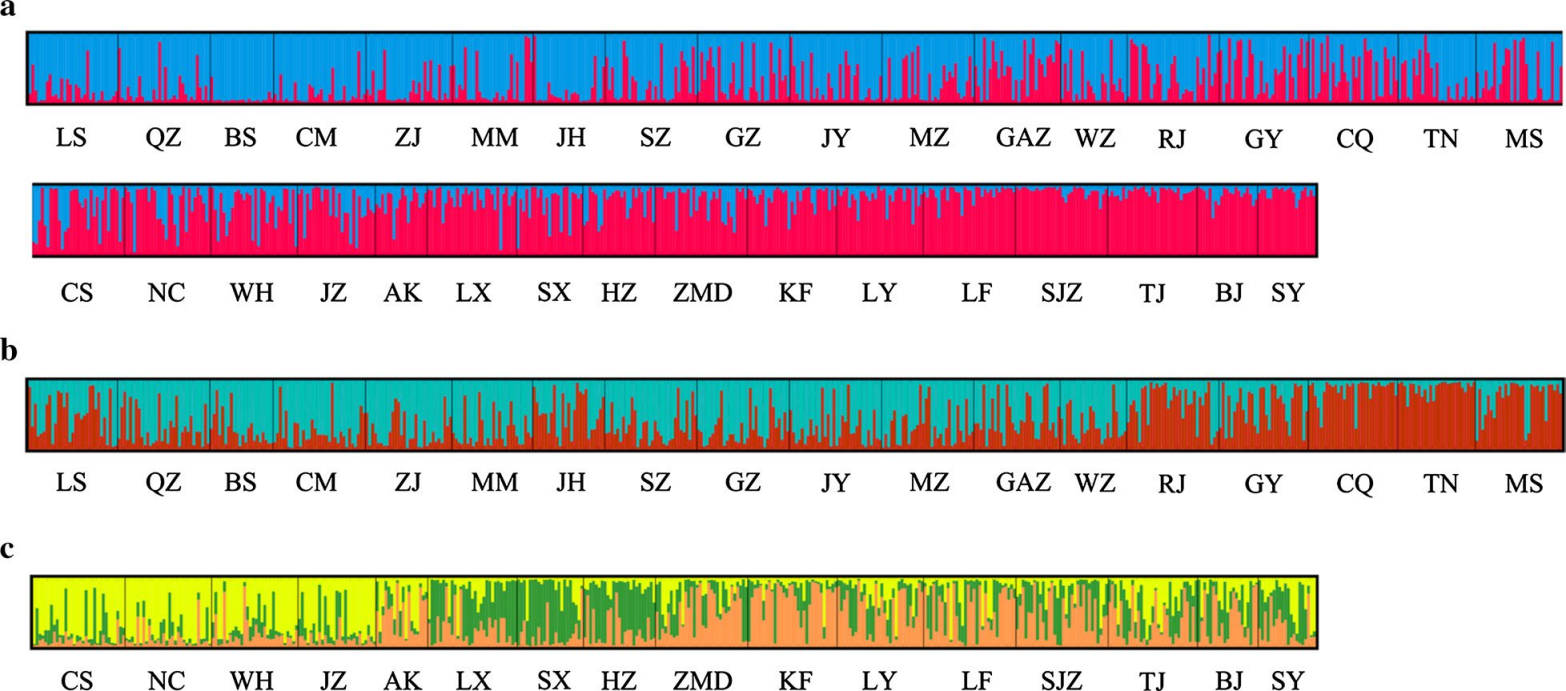
Genetic structure of *Ae. albopictus* within 34 locations. Each vertical bar in the plots represents an individual sample and each color represents a cluster, where the color of the bar indicates the probability of assignment to each of K optimal clusters (different colors) determined using Evanno et al.’s ΔK methods. **a** K = 2 for all populations. **b** K = 2 for 18 populations in southern and western areas. **c** K = 3 for 16 populations in central, eastern, and northern areas

**Fig. 3:**
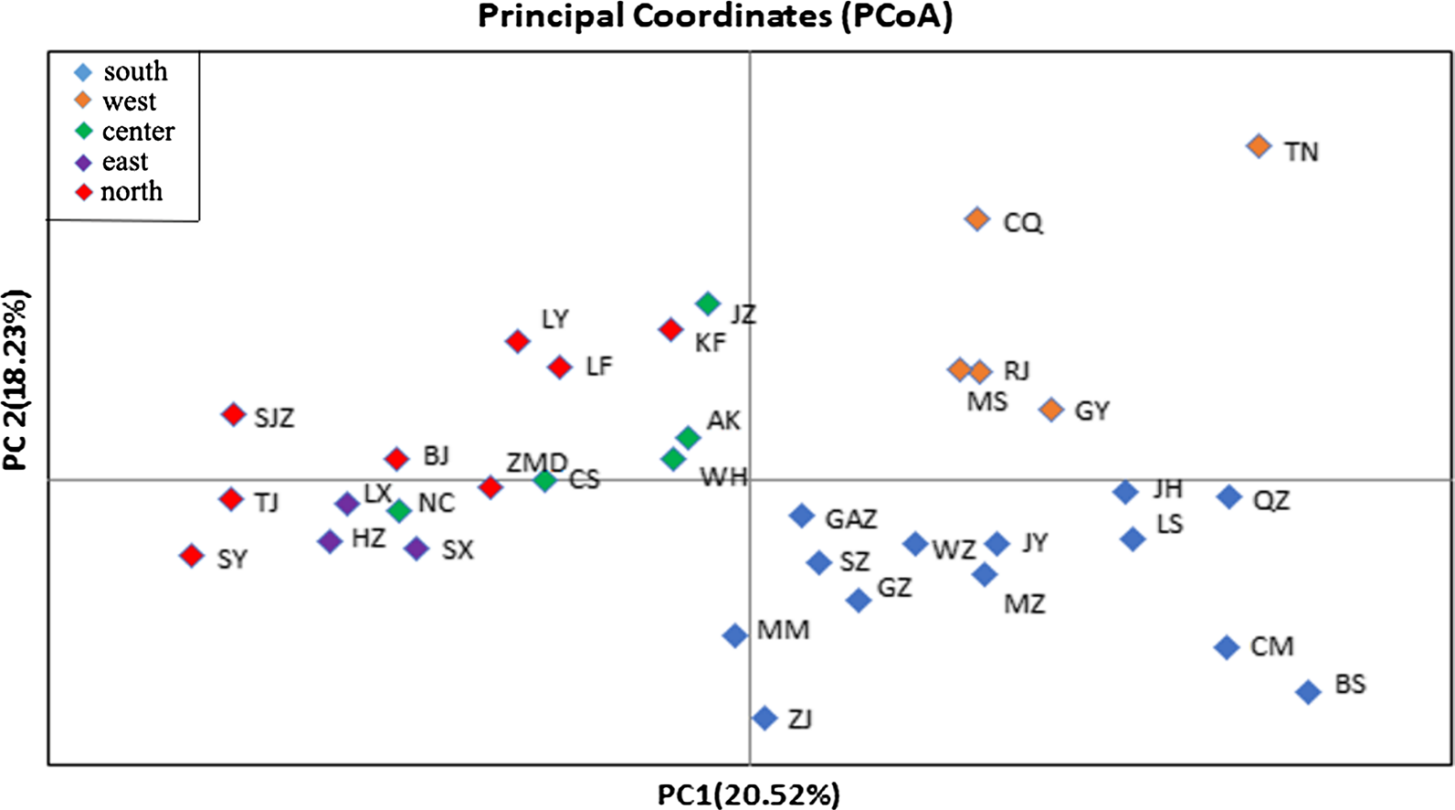
Principal coordinates analysis based on co-dominant genotypic genetic distance, displaying genetic similarities among populations of *Ae. albopictus* sampled from different regions in China

Based on Bayesian clustering analysis and the Delta K method, each population in this study was assigned to one of two genetically differentiated groups (K = 2, Additional file 7: Figure S2a), which splits southern and western China from a group comprising the eastern, central and northern populations (Fig. 2a). Some admixture was observed between the groups. A further independent Bayesian clustering analysis and higher resolution were obtained by analyzing the structure plot for the two groups. In the first group, the southern populations can be separated from the western populations at K = 2 (Fig. 2b, Additional file 7: Figure S2b). In the second group, the central, eastern, central northern, and northern clusters were divided at K = 3 (Fig. 2c, Additional file 7: Figure S2c). Additionally, the results of principal coordinates analysis (Fig. 3) illustrated the genetic similarities among different populations in each region, which was similar to the results from STRUCTURE. Based on these results, we divided the 34 populations into five clusters: southern (LS, QZ, BS, CM, ZJ, MM, JH, SZ, GZ, JY, MZ, GAZ, WZ), western (RJ, GY, CQ, TN, MS), central (CS, NC, WH, JZ, AK), eastern (LX, SX, HZ), and northern (ZMD, KF, LY, LF, SJZ, TJ, BJ, SY). The AMOVA results (Table 4) indicate that the majority of the variation in *Ae. albopictus* was found among individuals and individuals within populations, accounting for 83.16% and 14.21% of the variation, respectively, while variations among groups and populations within groups accounted for only 1.30% and 1.32% of the total variation, respectively. Fisher’s exact test showed that there was significant genetic variation at these four levels.

**Table 4:** Analysis of molecular variance of populations from seven different clusters

Source of variation	*df*	Sum of squares	Variance components	Percentage of variation	*P-*value	Fixation index
Among groups	4	121.507	0.060	1.30	*P* < 0.0001	*F*_CT_ = 0.013
Among populations within groups	29	250.076	0.061	1.32	*P* < 0.0001	*F*_SC_ = 0.013
Among individuals within populations	943	4837.406	0.653	14.21	*P* < 0.0001	*F*_IS_ = 0.146
Within individuals	977	3735.000	3.823	83.16	*P* < 0.0001	*F*_IT_ = 0.168
Total	1953	8943.989	4.597			

*Abbreviations*: *df*, degrees of freedom

**Fig. 4:**
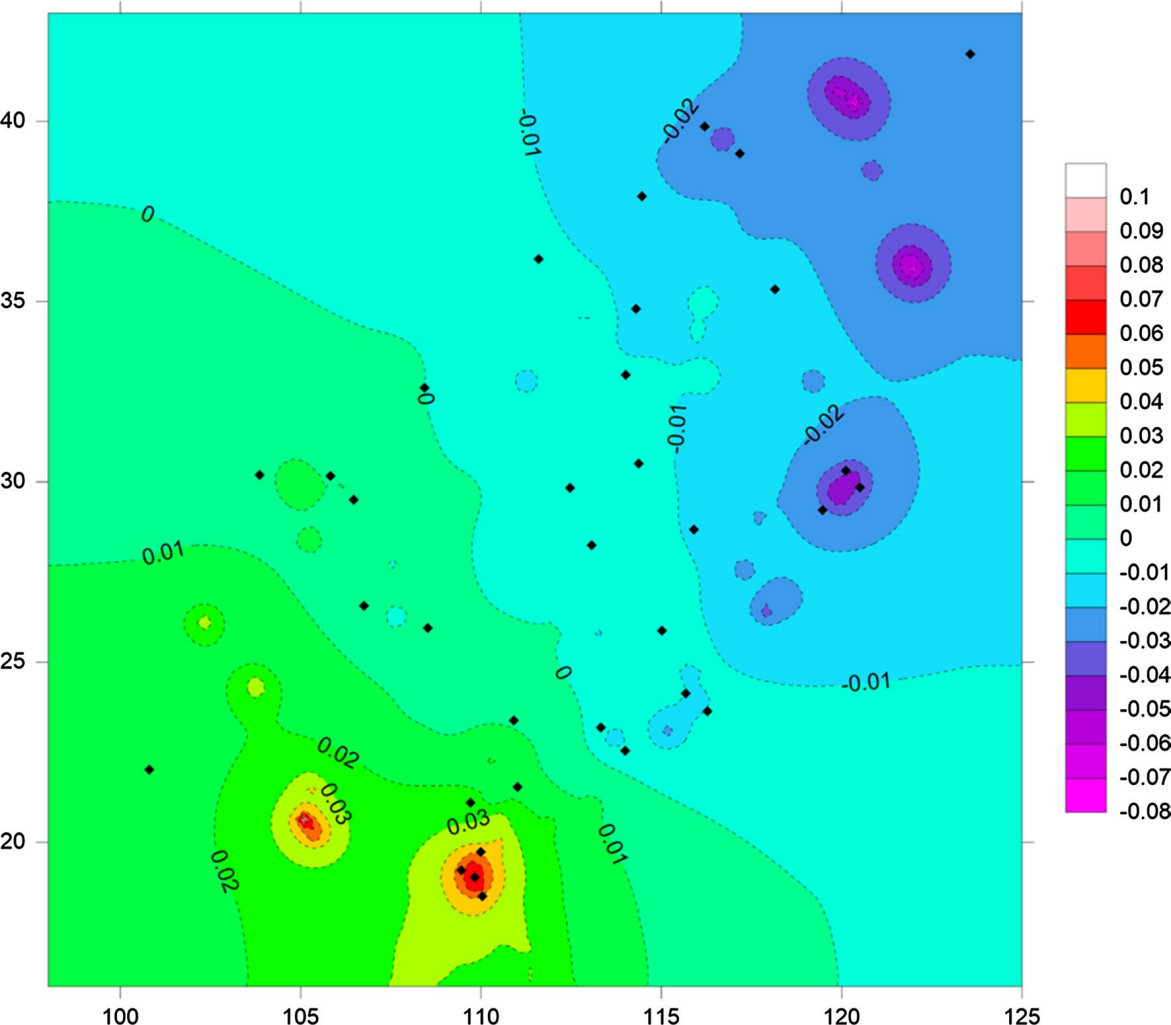
Genetic landscape shape plot showing patterns of spatial genetic distance for 34 populations of *Ae. albopictus*. The GLS interpolation analysis is shown in Surfer software, where X- and Y-axes correspond to geographical locations and the different colors of image regions represent genetic distances

**Fig. 5:**
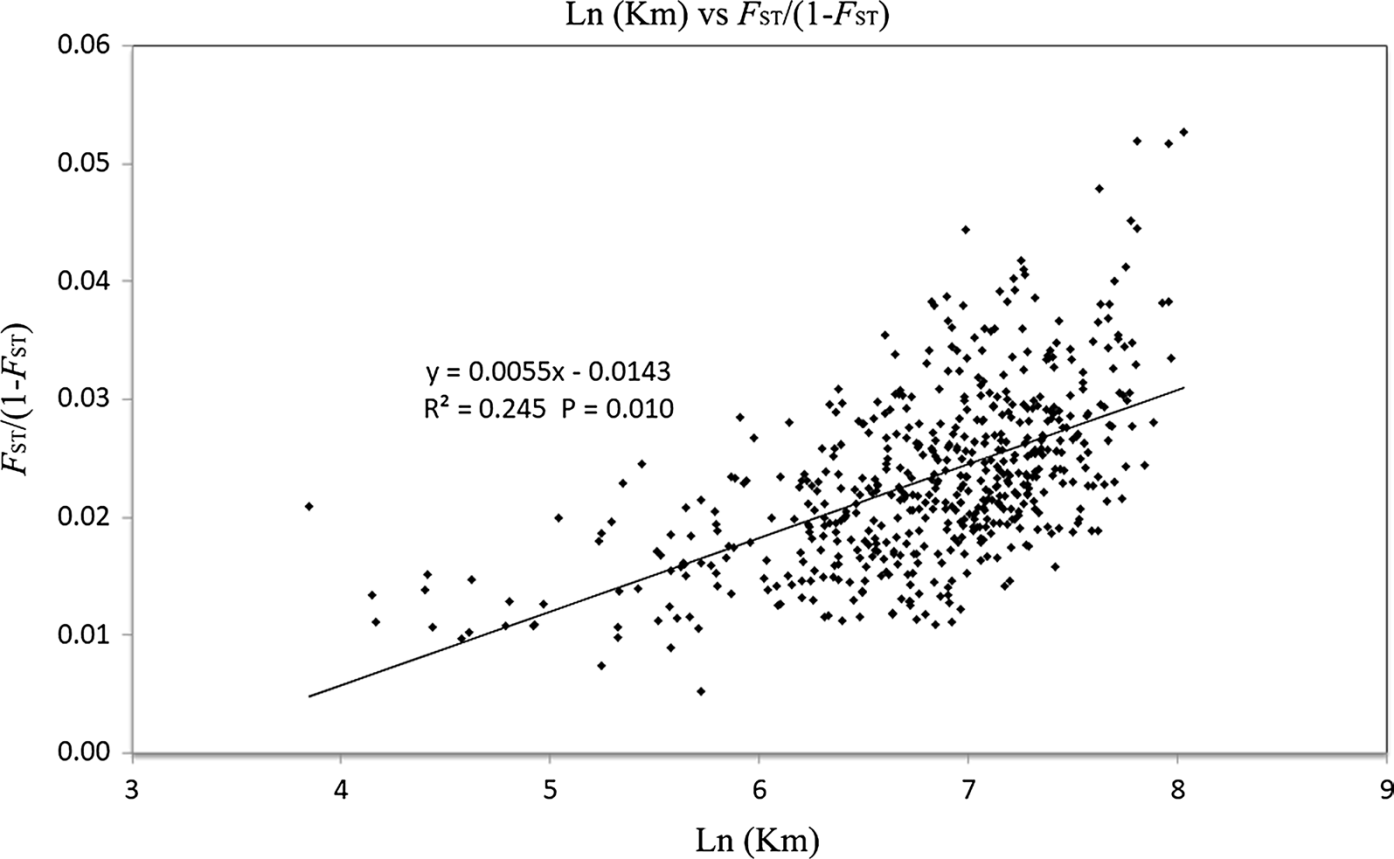
Correlation between genetic distance [*F*_ST_/(1 − *F*_ST_)] and geographical distance [Ln (km)] for all locations in China. The relationship was significant (Mantel test; *R*^2^ = 0.245, *P* = 0.01)

The pairwise *F*_ST_ values of *Ae. albopictus* ranged from 0.005 to 0.049, with 500 out of 561 (89.13%) *F*_ST_ values showing significant genetic differentiation and one losing the significance after Bonferroni correction (Additional file 8: Table S6). However, the gene flow calculated by *F*_ST_ showed that the minimum value of *Nm* between populations is 4.88 (Additional file 8: Table S6), suggesting strong gene flow. The genetic landscape shape analysis identified major potential gene flow barriers (Fig. 4). The high peaks of differentiation were found in southern and southwestern China, and low genetic differentiation was detected among populations in the eastern and northern regions (Fig. 4). The Mantel test showed statistically significant correlation (*R*^2^ = 0.245, *P* = 0.01) between the genetic distance (estimated as *F*_ST_/(1 − *F*_ST_)) and geographical distance (estimated as Ln (km)) between populations (Fig. 5, Additional file 9: Table S7).

### Correlation between population genetic indices and dengue incidence

In order to explore the association between genetic structure and vector-borne disease, we conducted Pearsonʼs correlation analysis of six genetic indices and annual dengue incidence from 2011 to 2015 in different provinces (Table 5). Allelic richness (*r* = 0.530, *P* = 0.020) and private allelic richness (*r* = 0.551, *P* = 0.015) showed a positive correlation with dengue incidence after log-transformations. However, the significance was lost after the Bonferroni correction for multiple testing (adjusted *P*-value < 0.00238). Shannon’s information index (*r* = 0.455, *P* = 0.051), effective alleles (*r* = 0.353, *P* = 0.139), observed heterozygosity (*r* = 0.210, *P* = 0.388) and inbreeding coefficient (*r* = 0.089, *P* = 0.717) showed no statistically significant correlation to dengue incidence after log transformations.

**Table 5:** The correlation of dengue incidence with six genetic indices among *Ae. albopictus* populations in different provinces

Province	Allelic richness	Private allelic richness	*Ne*	SI	*Ho*	*F*_IS_	Annual dengue cases^a^
Hainan	7.171	0.181	4.754	1.748	0.548	0.278	56
Guangdong	6.453	0.047	3.983	1.559	0.633	0.093	9541
Yunnan	7.263	0.418	4.718	1.682	0.592	0.204	1548
Guangxi	6.183	0.094	3.542	1.459	0.591	0.152	371
Guizhou	6.106	0.005	3.784	1.487	0.577	0.159	3
Chongqing	6.530	0.029	4.485	1.586	0.647	0.081	42
Sichuan	6.320	0.016	4.000	1.530	0.561	0.214	20
Hunan	5.506	0.025	3.539	1.359	0.518	0.209	37
Jiangxi	6.379	0.090	3.982	1.539	0.553	0.195	16
Hubei	5.994	0.052	3.949	1.490	0.546	0.213	23
Shanxi (west)	5.579	0.000	3.657	1.394	0.644	0.028	11
Zhejiang	5.704	0.129	3.678	1.409	0.628	0.018	59
Henan	6.134	0.094	3.981	1.530	0.658	0.059	17
Shandong	5.811	0.002	3.917	1.447	0.582	0.136	9
Shanxi (east)	5.697	0.011	3.785	1.422	0.551	0.183	1
Hebei	5.827	0.001	3.855	1.434	0.564	0.133	8
Tianjin	5.713	0.002	3.435	1.359	0.633	0.031	4
Beijing	5.653	0.000	3.455	1.379	0.600	0.076	69
Liaoning	4.904	0.001	3.183	1.236	0.597	0.001	7
Pearson’s correlation coefficient^b^	0.530	0.551	0.353	0.455	0.210	0.089	
*P-*value	0.020	0.015	0.139	0.051	0.388	0.717	

^a^From 2011 to 2015 (per 100,000,000 persons)

^b^The annual dengue case data was rescaled with a log transformation using the equation Ln (µ), where µ is the number of cases per 100,000,000 people

*Abbreviations*: *Ne*, effective number of alleles; SI, Shannonʼs index; *Ho*, observed heterozygosity; *F*_IS_, inbreeding coefficient

## Discussion

Few studies have reported the population structure and genetic diversity of *Ae. albopictus* in China, and they have focused on using mitochondrial DNA markers to analyze genetic diversity, regional structure, mainly in populations in southern China, or they have used a limited sampling size [31, 55]. For example, Guo et al. [31] sequenced the mitochondrial DNA cytochrome *c* oxidase subunit 1 (*cox*1) gene of 140 *Ae. albopictus* from 14 populations in southern China, and Zhang et al. [55] sequenced *cox*1 of 119 *Ae. albopictus* from 17 populations in China. The results of these studies showed low genetic diversity and shallow genetic differentiation between some of the study populations. In the present study we included many new study sites and populations, such as those in the northern (SY, SJZ, TJ), western (MS, TN), and central areas (WH, JZ), etc. The 34 collecting sites were widely distributed, providing comprehensive information on the population structure of *Ae. albopictus* throughout China. The correlation of average allele richness and sample size showed that when sample size < 15, allele richness increased rapidly as sample size increased, whereas it increased only slightly when sample size > 25. The number of samples in most populations was within the optimum range (25–30) [56]. The mean number of alleles per locus and the allelic richness were higher in southern populations than in northern populations, and the diversity of alleles gradually decreased from south to north. The climate in northern temperate areas may affect the life traits of *Ae. albopictus*. The period in which the climate is suitable for mosquito survival is longer in southern subtropical areas than in northern areas. The relatively low temperatures and dry climate in the north may not be suitable for mosquito survival, reproduction, and dispersal, resulting in lower allele richness and population diversity in northern populations [57, 58].

A major drawback of using microsatellite markers in population genetic studies is the potential impact of null alleles, such as reducing population genetic diversity and increasing genetic differentiation among populations [59, 60]. Microsatellite null alleles are commonly more frequent in arthropods than in other species [61–63]; this may be a result of large effective population size, as species with large effective population size have relatively large proportions of individuals with null alleles [64]. Many studies have determined that a null allele frequency < 0.2 has no significant effect on the accuracy of data analysis, and its effect on genetic diversity and genetic structure may be even less [64, 65]. In our study, null allele frequency was < 0.2 or absent, except at locus Aealbmic12. Expected heterozygosity is a common parameter for measuring the genetic polymorphism of a population [66]. Takezaki & Nei [67] suggested that an expected microsatellite heterozygosity between 0.5–0.8 indicates that the population genetic polymorphism is high. In the present study, the expected heterozygosity in all populations was between 0.6–0.8, and most were higher than the observed heterozygosity, indicating a deficit of heterozygosity except in the SZ and SY populations [68]. Insecticides have been used frequently in some areas, and fragmentation of habitats can intensify inbreeding within small populations, leading to a heterozygosity deficit. For bottleneck testing, we used the two-phase mutation (TPM) model, which is considered the best-fit for microsatellite data [44]. Our bottleneck test results indicated that only the SJZ population may have experienced a temporary bottleneck; this may be related to the wide use of insecticides [69] or sharp climatic changes [70] in the local area causing a decline in local populations.

Our results showed that *F*_ST_-values in all pairwise populations were not high, but most were significantly different, indicating genetic differentiation between populations. The low genetic distance and pairwise *F*_ST_ may be related to the fact that most of the study sites are central cities in their provinces and transportation between them is convenient. Jinghong, a busy tourist destination close to the Southeast Asian countries, connects to many cities *via* highways and airways but has no trains or high-speed rail, which explains the relatively high pairwise *F*_ST_ between Jinghong and other cities. Low *F*_ST_-values with no significant difference may be attributed to the close distance, convenient transportation, high traffic, and similar environmental conditions between populations [71]. China is home to an ancestral population of *Ae. albopictus*, and most populations in China have been stable for a long time [22]. *Nm* > 1 indicates that a population is sufficient to prevent the occurrence of genetic differentiation [72]. Our results showed strong gene flow between populations, which can reduce genetic differentiation. The results of genetic relatedness showed the individuals among different collection sites with long distance had different degrees of kinship, indicating potential human-aided dispersal of *Ae. albopictus* across the country. Extensive mosquito dispersal, probably aided by human transportation between these regions, also helps explain the low differentiation and the possible clustering between different regions.

There is some limitation for microsatellites compared to SNPs, the third generation DNA markers. Weaker separation between populations was usually obtained with microsatellites compared to SNPs. A total of 934 SNPs showed stronger power in distinguishing closely related individuals at a small spatial scale than 8 microsatellites in *Ae. aegypti* populations [73]. A total of 2185 SNPs outperformed nine highly variable microsatellites in parentage and kinship assignment in *An. darlingi* populations, and the conventional approaches based on microsatellites may underestimate overall genetic distances in closely related vector populations [74]. The pairwise *F*_ST_ or the degree of differentiation in the present study may be smaller for microsatellites than if SNPs have been used. Therefore, we might overestimate the gene flow between populations through the analysis of microsatellites. Mitochondrial DNA has been widely used for the study of molecular taxonomy, phylogenetic relationships and population genetics in mosquitoes [75–77]. However, microsatellites outperformed mtDNA in assessing spatial genetic structures. Only microsatellites not mtDNA showed small but positive and significant isolation-by-distance patterns in *An. sinensis* populations [78]. No genetic structure was detected through mtDNA data while a high level of genetic structure was detected using the microsatellite markers inside polymorphic inversions in *An. arabiensis* populations [79]. Guo et al. [31] discovered the cryptic species in Wuzhou using mitochondrial DNA cytochrome *c* oxidase subunit 1 (*cox*1) gene to analyze population genetics of *Ae. albopictus* in southern China. We also have collected *Ae. albopictus* population from a different village in Wuzhou, but we did not find any individual belonging to a cryptic species by *cox*1 sequencing. One of the possible reasons might be due to location-specific or seasonal abundance features of the cryptic species.

The spread of dengue fever has been affected not only by the climate (e.g. temperature, rainfall, relative humidity and sunshine) [80, 81], but also by the vector indices (e.g. container, house, Breteau, pupal or adult indices) and genetic factors of the populations [82–84]. *Aedes aegypti* population from French Polynesia was more susceptible to infection and had higher ability to transmit DENV-1 than *Ae. aegypti* population from New Caledonia in the same experimental environment [84]. The genetic background of mosquito populations could influence the vector competence of mosquitoes, and the genetic indices can be utilized as a potential predictor of local dengue epidemiology [85]. The six genetic indices are the main indices of population genetics, which could directly reflect the actual genetic variability and diversity of local mosquitoes. But they were not significantly correlated with dengue incidence during 2011–2015. One possible reason might be that dengue outbreaks are mainly affected by climate and environment rather than genetic background of *Ae. albopictus* population in China. The other one might be that dengue incidence is associated with other genetic indices or other genetic markers rather than the six indices from 13 microsatellites in this study. One genetic index, the inbreeding coefficient (*F*_IS_) might be expected to show significant positive correlation to dengue incidence, due to the vertical transmission of dengue virus to mosquito offspring [86, 87]. This natural transovarial transmission process has been demonstrated in field-collected *Ae. aegypti* mosquitoes from some different areas [88–90]. The rate of vertical transmission initially increased in the few generations (F1–F2) of *Ae. aegypti* mosquitoes, and in subsequent generations it was found to be steady until at least the 7th generation through the inbreeding of each generation [91]. We inferred that the inbreeding within the mosquito population might lead to a substantial number of offspring carrying the dengue virus and an increased disease transmission within local areas. Inbreeding coefficient has been found to be positively associated with dengue incidence in *Ae. aegypti* populations [85]. However, our study detected no significant correlation. One plausible explanation is that the amount of viral infection in the mosquitoes collected in our study is not enough to spread vertically to offspring. In addition, the endosymbiotic bacteria *Wolbachia* existing in *Ae. albopictus*, not in *Ae. aegypti* naturally, has a blocking effect on the vertical transmission of dengue virus [92, 93]. Although the specific mechanism of *Wolbachia* inhibiting dengue virus is unclear, the cytoplasmic incompatibility (CI) induced by *Wolbachia* can reduce the mosquito populations [94], and *Wolbachia* can induce density-dependent inhibition to dengue virus in mosquito cells [95]. At present, mosquitoes transfected with *Wolbachia* have been applied in the control of mosquitoes and mosquito-borne diseases [96, 97]. The application would change the population structure and genetic indices of local mosquitoes, and it would require our monitoring. Further research is needed to ascertain on whether these genetic indices could be supplementary indices for the prediction of the local dengue epidemiology.

## Conclusions

This study reported not only the spatial genetic structure of *Ae. albopictus* but also the correlation of genetic indices with dengue incidence. Our results may have implications for predicting future dengue outbreaks and understanding the epidemiology, prevention and control of vector-borne diseases. Strong gene flow probably assisted by human activities inhibited population differentiation and promoted genetic diversity among *Ae. albopictus* populations. This may represent a potential risk of rapid spread of mosquito-borne diseases. The data collected in this study, combined with other related factors such as climate, rainfall and human activities, will be valuable for vector control efforts as well as epidemiological prediction and modeling of the incidence and spread of vector-borne diseases.

## Supplementary information

**Additional file 1: Table S1.** The *Wolbachia* infection status in populations.

**Additional file 2: Table S2.** The pairwise genetic relatedness between individuals.

**Additional file 3: Table S3.** Analysis of Hardy–Weinberg equilibrium.

**Additional file 4: Table S4.** Analysis of linkage disequilibrium.

**Additional file 5: Table S5.** Genetic indices of 34 populations.

**Additional file 6: Figure S1.** Average allele richness of microsatellite loci in 34 populations.

**Additional file 7: Figure S2.** Scatter plots of Log probability of the data (Left) and Delta K (Right) for *Ae. albopictus* populations. Delta K plots are based on the rate of change in the log probability of the data between successive K values. **a** All populations. **b** 18 populations in southern and western areas. **c** 16 populations in central, eastern, central northern, and northern areas.

**Additional file 8: Table S6.** *F*_ST_ and *Nm* between 34 populations.

**Additional file 9: Table S7.** Genetic distance and geographical distance between 34 populations.

## Data Availability

The data sets supporting the results are included within the article and its additional files.

## Abbreviations

AMOVA: analysis of molecular variance
PIC: polymorphic information content
SI: Shannonʼs index
HWE: Hardy–Weinberg equilibrium
LD: linkage disequilibrium
