# Supplementary figures and images for: Patterns of spatial genetic structures in Aedes albopictus (Diptera: Culicidae) populations in China
Source: Parasit Vectors. 2019 Nov 21;12:552. doi: 10.1186/s13071-019-3801-4 (PMC6873696; doi:10.1186/s13071-019-3801-4)

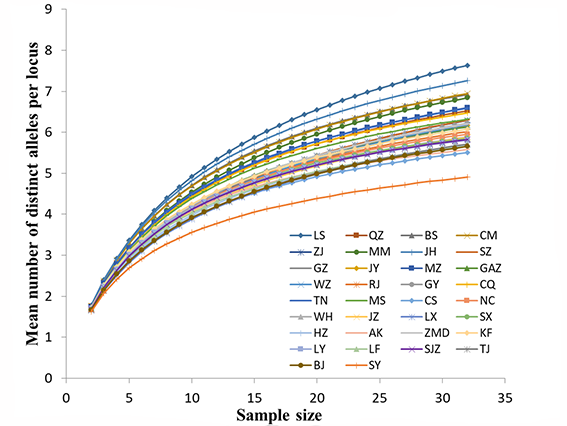

Supplement: Supplementary file 6 — Additional file 6: Figure S1. Average allele richness of microsatellite loci in 34 populations. [file 13071_2019_3801_MOESM6_ESM.tif]

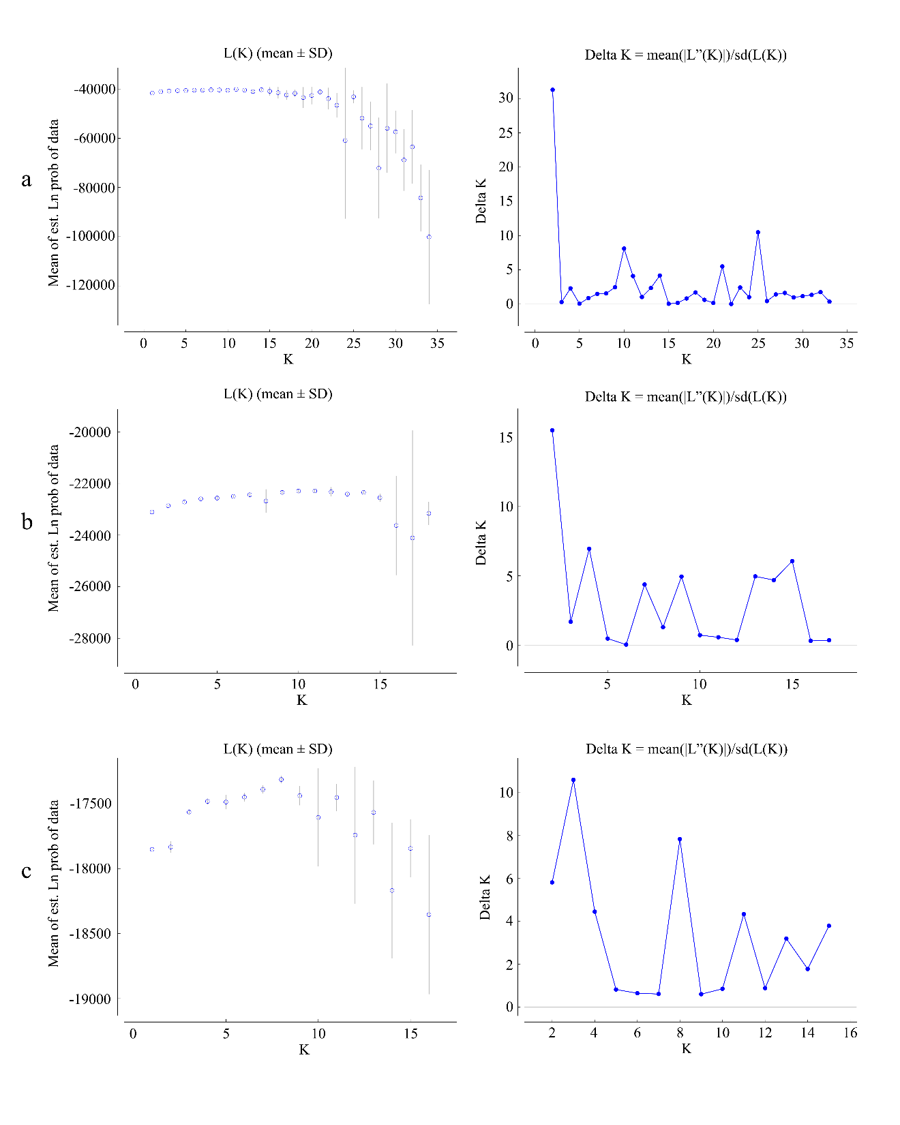

Supplement: Supplementary file 7 — Additional file 7: Figure S2. Scatter plots of Log probability of the data (Left) and Delta K (Right) for Ae. albopictus populations. Delta K plots are based on the rate of change in the log probability of the data between successive K values. a All populations. b 18 populations in southern and western areas. c 16 populations in central, eastern, central northern, and northern areas. [file 13071_2019_3801_MOESM7_ESM.tif]
